# A Qualitative Exploration of Unanticipated Effects Experienced By People With Chronic Obstructive Pulmonary Disease Who Participated in Interventions Designed to Reduce Sedentary Behaviour

**DOI:** 10.1111/hex.70694

**Published:** 2026-05-24

**Authors:** Fiona Coll, Vinicius Cavalheri, Daniel F. Gucciardi, Kylie Hill

**Affiliations:** ^1^ Curtin School of Allied Health, Faculty of Health Sciences, Curtin University Perth Western Australia Australia; ^2^ South Metropolitan Health Service Allied Health Perth Western Australia Australia; ^3^ Exercise Medicine Research Institute Edith Cowan University Perth Western Australia Australia; ^4^ Department of Physiotherapy Sir Charles Gairdner Hospital Perth Western Australia Australia

**Keywords:** behaviour change techniques, chronic obstructive pulmonary disease, sedentary time

## Abstract

**Introduction:**

This study explored the lived experiences of people with chronic obstructive pulmonary disease (COPD) who participated in interventions designed to reduce the accumulation of sedentary behaviour in prolonged uninterrupted bouts.

**Methods:**

Ten participants with stable COPD participated in a multi‐component intervention that spanned over 9 months and was designed to address the intervention message ‘If you have been sitting or resting continuously for 30 min, stand up and move’. Interventions comprised participation in an 8‐week programme of supervised exercise training coupled with behaviour change techniques. Throughout the intervention period the person delivering the intervention kept detailed notes of their interactions with each participant. On completion of the intervention period, participants completed a semi‐structured interview that was transcribed verbatim, and these data were analysed and coded to generate themes.

**Results:**

These phenomenological data are presented as narrative case studies. Several unanticipated benefits were described which were summarised into three themes: (1) released from a state of feeling completely trapped, (2) reconnected with meaningful activities of their past self and (3) reclaimed agency and autonomy.

**Conclusions:**

A multicomponent intervention that was designed to reduce the accumulation of sedentary behaviour in prolonged uninterrupted bouts had several unanticipated benefits pertaining to the way people perceived themselves as individuals. The effects are unlikely to be fully captured or appreciated using quantitative methods alone. This study highlights the importance of understanding the effect of interventions and therapeutic interactions through the lens of the person living with the chronic condition.

**Patient or Public Contribution:**

The participants and researchers collaborated to co‐design the study's intervention which included personalised behaviour goals to reduce sedentary behaviour. This working partnership between participants and researchers utilised interviews, diary entries and group focus sessions to construct tailored interventions. Our findings showed unanticipated benefits for participants rather than objective change in reducing sedentary behaviour. Therefore, clinicians may consider these experiences by people with COPD as an important outcome.

## 1 Introduction

Chronic obstructive pulmonary disease (COPD) is a complex multifactorial disease and one of the leading causes of mortality and morbidity worldwide. In people with COPD, the two most debilitating symptoms are dyspnoea and fatigue [1, 2]. To help mitigate their experience of these symptoms, people with COPD adjust their lifestyle and become less physically active and increasingly sedentary [3]. A reduction in physical activity has been shown to occur early in the disease and as their condition progresses, many people with COPD have difficulty completing activities of daily living [2].

There are several interventions that can reduce the experience of dyspnoea during daily life and these are often recommended as a standard of care in national and international guidelines for the management of COPD [4]. Nevertheless, the effect of these interventions on participation in daily physical activity is unclear. Specifically, a review of 76 studies that explored a range of interventions such as supplemental oxygen therapy, physical activity counselling, bronchodilator therapy and exercise training found an absence of any high‐quality evidence to support the effect of any intervention on measures of physical activity [5].

Since 2010, in people with COPD, there has been interest in whether or not it is more realistic to target reductions in sedentary time rather than increases in physical activity [6]. This refocus has been justified as breaking up periods of sedentary time (ST) with light intensity physical activity (LIPA) (such as standing and walking) is likely to produce less symptoms than asking people with COPD to engage in moderate to vigorous physical activity [7]. In the general population, there is convincing evidence that specifically targeting reductions in ST confers health benefits [7, 8]. We also now understand the physiological mechanisms underpinning these effects [8]. These mechanisms that drive the association between increasing ST and the risk of cardiovascular and metabolic disease appear to be the metabolism of skeletal muscle and how it utilises triglycerides and glucose [9]. An accumulation of prolonged uninterrupted bouts of ST lowers skeletal muscle activity to siphon off and use triglycerides for energy [9]. Animal models suggest that lipoprotein lipase activity can be restored with participation with LIPA rather than moderate to vigorous physical activity [9]. Epidemiological studies have demonstrated that cardiometabolic health can be optimised by reducing ST, especially ST accumulated in prolonged uninterrupted bouts [10]. A study in 2017 of older adults (*n* = 7995), participants that measured ST using an accelerometer every 6 months over 6 years suggested that accumulating ST in bouts less than 30 min was associated with less of an increased risk of all‐cause mortality than other patterns of accumulation [11].

Due to the evidence of cardiometabolic risk with sedentary behaviour in the general population, we completed a single‐case experimental design study in which a group of 10 participants with stable COPD interacted with a clinical‐researcher over a 9‐month period with the goal of reducing prolonged uninterrupted bouts of ST by engaging in LIPA. In this paper, we present the results of qualitative work which sought to understand the persons lived experience with engaging in this study.

## 2 Research Question

What were the lived experiences of people who participated in a multicomponent intervention comprised of a PRP augmented by BCT that focused specifically on reducing the accumulation of sedentary behaviour in prolonged uninterrupted bouts?

## 3 Methods

The study was approved by the relevant Human Research Ethics Committees (HREC) to allow recruitment from four hospitals across Perth, Western Australia; Bentley Health Service approval number research governance system (RGS)3353, Joondalup Health Campus; approval number HREC1915, Rockingham General Hospital approval number RGS3353 and Royal Perth Hospital approval number RGS3353. Reciprocal ethics approval was obtained from the Human Research Ethics committee at Curtin University (HREC2019‐0429). Data collection took place between September 2019 and March 2022.

All participants provided written informed consent prior to data collection. The study has been reported in accordance with Standards for Reporting Qualitative Research checklist see Appendix A.

### 3.1 Study Design

We completed a single‐case experimental design study in which a group of 10 participants with stable COPD interacted with a clinical researcher with the goal of reducing prolonged uninterrupted bouts of ST by engaging in LIPA. Ther intervention we applied composed of multiple components including an 8‐week pulmonary rehabilitation programme (PRP) and the application of individiualised behaviour change techniques (BCTs) informed evidence collated within the systematic reviews and our own pilot work. Although we did not see changes in objective measures of ST collected using an activPAL monitor (PAL Technologies Ltd., Glasgow, UK), detailed qualitative data were collected which sought to understand the persons lived experience with engaging in this study. These analyses reveal the way people with COPD made sense of this intervention.

### 3.2 Participants

Participants were recruited to the study if they: (i) were aged ≥ 40 years, (ii) had a primary diagnosis of COPD, (iii) had been referred to participate in an outpatient hospital‐based PRP, and (iv) had access to a Smartphone. Exclusion criteria comprised: (i) self‐reported musculoskeletal or cardiac condition that precluded them from engaging in LIPA, (ii) prescribed ambulatory oxygen or walking aid (e.g., rollator frame), and/or (iii) limited understanding of the English language. Although participants with a recent moderate to severe exacerbation of COPD were eligible for inclusion, participation was delayed until they were 4 weeks post‐exacerbation. Exacerbations were defined as a worsening of symptoms for the last 48 h that were beyond normal day to day variability. Exacerbations that could be managed by the person at home were considered ‘mild’. Those that required an unscheduled visit to a general practitioner were considered ‘moderate’. Those that required presentation to a hospital were considered ‘severe’ [12].

### 3.3 Intervention

The intervention period was 9 months (36 weeks) in duration over which time the intervention message was kept consistent for each participant; ‘If you have been sitting or resting continuously for 30 min, stand up and move’. The intervention components were formed by psychology and behavioural science literature [13], data from systematic reviews [14, 15, 16] and consumer feedback sought during a pilot study. Central to this framework is the capability, opportunity and motivation (COM‐B) system [17], which underpins the mechanism by which behaviour is enacted. This approach was particularly pertinent was important given our focus on delineating the features that influenced decision‐making processes related to reducing the accumulation of ST in prolonged uninterrupted bouts. The components comprised an 8‐week PRP together with BCTs that were selected from an established BCT taxonomy (v1) that included 93 hierarchically‐clustered techniques [18]. The choice of BCTs were individualised for each participant with a focus on promoting self‐efficacy and independent decision making to achieve the intervention message [18]. Social Cognitive Theory was used in conjunction with BCTs focusing on self‐efficacy, observational learning and to enhance BCTs such as in social support (unspecified), instruction in how to perform the behaviour and feedback on behaviour [13]. A summary of the BCTs used and an example of the way they were implemented in the study are presented in Table 1.

**Table 1:** Summary and examples of the behaviour change techniques used.

	Behaviour change techniques	Example
1.1	Goal setting (behaviour).	The participant was asked to nominate a goal to reduce ST such as stand up from sitting every 30 min and walk around the house in the evening while watching TV.
1.4	Action planning.	The participant was prompted to plan the action, the intervention time and the day. For instance, stand up from sitting every 30 min and walk around the house while sitting watching TV on Tuesday to Friday between 6 pm and 10 pm.
1.5	Review behaviour goal(s).	The participant and the clinician‐researcher reviewed the goals set from the previous visit. These goals may need to be increased or reduced depending how the participant achieved the previous goal. For instance, to progress the goal of reducing sitting during watching TV the experimental intervention window is progressed, stand up from sitting every 30 min and walk around the house while watching TV on Tuesday to Friday between 5 pm and 10 pm.
2.2	Feedback on behaviour.	The participant was informed by the clinician‐researcher via SMS texts after the agreed experimental intervention window and/or face to face sessions of how many times sitting time was interrupted and for how long (as recorded on their Fitbit). This information was a discussion point that promoted 1.5 review behaviour goal(s).
2.7	Feedback monitoring outcome(s) of behaviour.	The participant was given specific feedback by the clinician‐researcher (who remotely monitored the participant's Fitbit) via SMS texts after the experimental intervention occurred and/or face to face, whether this was achieved, ST was reduced by standing up from sitting every 30 min.
3.1	Social support (unspecified).	The participant may have a support person who was recruited as part of the study or a person who was called upon by the participant to support them in reducing ST. For instance, a neighbour who encouraged the participant go for a short walk with them.
4.1	Instruction on how to perform the behaviour.	The clinician‐researcher. instructed participants on how to break up sitting by either standing, walking around the house or getting up for a drink of water.
5.1	Information about health consequences.	Verbal and written education of the health risks of prolonged uninterrupted bouts of ST were provided and how this can be reduced by replacing sitting with LIPA.
8.2	Behaviour substitution.	The participant was encouraged to stand up from sitting and walking around the house, therefore substituting ST with LIPA.
8.3	Habit formation.	After dinner at 7 pm the participant will sit and watch TV until 10 pm. The participant receives an SMS text at 7 pm from the clinician‐researcher with the message, ‘while watching TV stand up from sitting every 30 min’.
8.4	Habit reversal.	The participant stands from sitting and walks to the kitchen to make a cup of tea for others in the house rather than having the cup of tea made for them, while sitting watching TV.
8.6	Generalisation of a target behaviour.	The participant had been performing upper limb strength exercises with free weights during the PRP class. Now they perform the exercises when they stand up from sitting as the weights were located by the TV.
8.7	Graded tasks.	Initially in the study the participant started by standing up from sitting every 30 min and this was progressed to standing up from sitting each time there was a TV advertisement.
11.1	Pharmacological support.	The participant was encouraged to use their Ventolin inhaler prior to target activity (e.g., playing bowls), and to carry the Ventolin for future potential use.
12.1	Restructuring the physical environment.	The participant planned to sit on the end of an aisle in a Bowling Club annual general meeting so as they have the potential to stand up regularly without disturbing the annual general meeting.
12.5	Adding objects to the environment.	The participant hung a picture of a champion tennis player on the wall of the toilet as a cue to stand up from sitting and go for a walk around the house.
12.6	Body changes.	The participant was prompted to strengthen upper and lower limbs so to assist in standing from sitting from a chair.
13.2	Framing/reframing.	The participant was encouraged to be more active or find an activity they enjoy so as to further reduce ST.
15.3	Focus on past success.	The participant and the clinician‐researcher reflected on days where the goal of standing up from sitting every 30 min over a 4‐h period was achieved, or when the participant reached their step count goal for that day.

Abbreviations: LIPA, light intensity physical activity; PRP, pulmonary rehabilitation programme; SMS, short messaging service; ST, sedentary time; TV, television.

The intervention was delivered over three Phases. Phase 1 took place between weeks 1 and 8 during which participants engaged in a PRP together with an intensive behaviour change intervention that was delivered as individual 30 to 45 min face to face sessions once a week. Participants were supplied a Fitbit and this device was paired to the Fitbit application on the participant's Smartphone. During these sessions the participant and the clinician‐researcher, a female physiotherapist and researcher, co‐designed personalised behaviour goals consistent with the intervention message related to reducing long uninterrupted bouts of ST and replacing this with LIPA. An example goal was:

‘Between 4 pm and 10 pm Each weekday, when you watch TV, stand up and get a glass of water/walk around the garden every time there is an advertisement’

Phase 2 took place between weeks 9 and 13, and was focused on promoting agency regarding strategies to achieve the intervention message. During this time, weekly face to face sessions continued, but rather than having the participant interact with the clinician‐researcher at the site of their PRP, the clinician‐researcher visited the participant in their local community (e.g., local park or shopping centre). Phase 3 took place between the weeks of 14 and 36 and was designed to allow the participant to reach complete independence regarding strategies to achieve the intervention message. During this Phase, the clinician‐researcher visited the participant once a month at their home or their local community. We prioritised the BCT of action and coping planning during periods of exacerbation or set‐backs. In addition to the one‐on‐one sessions, at some point during Phase 2 or 3, participants were invited to attend a single group session. These sessions were semi‐structured and participants were asked to share their experiences of the study and to brainstorm strategies that were helpful in reducing the accumulation of sedentary behaviour in prolonged uninterrupted bouts. These sessions also fostered connections between participants with similar lived experiences of COPD.

### 3.4 Qualitative Data Collection and Analysis

Qualitative data were collected in two ways. First, the clinician‐researcher diarised the main themes of each one‐on‐one discussion session with the participant. The clinician‐researcher had lengthy and meaningful engagements with each participant over the course of the intervention. The participant was also encouraged to diarise their experiences throughout the study these reflections were shared with the clinician‐researcher as a point of reference for discussion during the intervention sessions. At times during these sessions the participant and clinician‐researcher shared their diary entries which allowed for fairness, appropriateness and believability which sparked a richer and deeper analysis of the qualitative data. On completion of the study, these sessions were transcribed verbatim.

Second, on completion of the study, participants were invited to complete a semi‐structured exit interview (see Appendix B). The interview lasted 45 min and was completed face to face in a location of the participant's choosing, which was usually in their home. It was audio‐recorded and transcribed verbatim. The interview followed a guide, with probing questions that aligned with the behaviour change wheel, for all transcriptions, the participants and related people were assigned an alias to preserve their anonymity.

The study used a phenomenological approach as this explores the individuals' lived experiences and the meaning they attribute to those experiences [19]. Qualitative data collected during the initial semi‐structured interview (see Appendix B) before the intervention commenced and ongoing sessions with diary entries were used to shape each participant's intervention which allowed the participant to experience being central in their own study experience.

The analyses consisted of the clinician‐researcher first spending time familiarising herself with the data by reading and re‐reading the transcripts. She then coded the data and started to generate themes and this was done in Microsoft word. Interviews were analysed from a critical realism ontological position. During this analysis process, she reported her thoughts to ‘critical friends’ who were experienced practitioners (K.H., V.C. and D.G.). The ‘critical friends’ were not involved in the delivery of the intervention therefore had an outsider perspective, and therefore could challenge her to reflect upon and examine alternative ways to find meaning in the data. The purpose of the meetings was also to allow for transparency and contextual reflexivity so as to question potential subjectivity from the clinician‐researcher's interpretation of the participant's experiences. The qualitative data from the detailed notes and semi‐structured interviews were combined to (i) triangulate data where different methods were employed to seek collaboration of an underlying meaning of the data [20], (ii) explore the unconstrained reflections of participants' experiences of the programme [20, 21], and (iii) permit opportunities for different pathways of exploration for a more comprehensive deeper answer to research questions [21].

## 4 Results

Thirteen participants were recruited to the study. Three participants withdrew their consent to participant in the study due to difficulties ranging from mental health issues (n = 1) to the inability to fully commit to a long‐term study (n = 2). Two participants delayed the start of Phase 1 due to a moderate exacerbation of COPD and commenced the study when they were 4 weeks post‐exacerbation. Ten participants completed the study, see Appendix C Table C1 for participant characteristics. Of the 10 participants 7 (70%) identified themselves as male, see Appendix C Table S1 Two out of the 10 participants also had a loved‐one enrol in the study. Three common themes were generated that captured the unanticipated benefits of participating in this study. The term ‘unanticipated’ was employed purposefully to reflect our missed opportunity to incorporate outcome measures that tapped into these experiences, compared with the traditional ‘objective’ type metrics which are among the ‘modus operandi’ for most clinical and medical research. To highlight the lived experiences of participants in this study, three exemplars have been presented as narrative case studies. Themes supported by quotes from the participants' experiences. The three themes were as follows:

1. *‘Released from a state of feeling completely trapped’*

Chronic illnesses and conditions are known to engender states in which people feel trapped or prevented from participating fully in life. Participants described this state of feeling physically and emotionally trapped as largely characterised by a sense of shame. They perceived themselves and believed others also perceived them as being inadequate. For participants in the current study, these perceptions were triggered by their diagnosis of COPD and reinforced primarily by the lived experience of symptoms of the disease as well as perceived social stigma that they had caused their disease (e.g., by being a heavy smoker). This experience of feeling shame or anticipation of shortness of breath was the primary symptom that prevented participants from engaging in meaningful activities that required some degree of exertion (e.g., going to the local park with their family to play with their grandchildren). In turn, the decision to disengage from effortful activities reinforced feelings of anxiety and depression because of the fear of triggering an episode of dyspnoea. Participants described their experiences with our intervention as one that triggered a re‐interpretation of their physical barriers and disease mindset, thus enabling them to release themselves from their state of feeling completely trapped by their disease.

One of the participants, Alex a 58‐year‐old man, had stable COPD Global initiative for chronic Obstructive Lung Disease (GOLD) grade score of 2 [22], and Body‐mass index (BMI), airflow Obstruction, Dyspnoea, and Exercise capacity (BODE) [22] score of 2. Alex enroled in the study in his second year of living in his unit near the beach. Alex had felt shame and stigma with his diagnosis of COPD as he had been an ex‐smoker (60 pack‐years) and, therefore, was reluctant to tell people due to the potential of a negative response. Alex found shame that his dyspnoea made him fearful, but he also felt shame due to people's potential judgement of him because of his past heavy smoking causing a diagnosis of COPD. He interpreted people's concern or fuss if he became dyspnoeic as embarrassing, intrusive and a negative judgement of his life choices leaving him to feel trapped with his diagnosis. The emotions of fearfulness and the stress of scrutiny by others caused Alex to avoid walking at the beach. However, since he participated in the study he began to walk outside the unit with a carefree attitude ‘I don't care anymore’. Importantly, Alex now re‐interprets the ‘fuss’ people made of him when they witnessed his shortness of breath as caring and supportive rather than judgemental.

**Alex:** ‘Then it was getting (being short of breath) embarrassing. Sometimes if you are gasping for breath and you getting on the Ventolin and people start making a fuss based on people making judgement or whatever. And that's what stopped me from doing it (walking down the beach). But now I just walk, and I don't really care anymore. Yeah, that's what it is. If anything, I should be more appreciative that people care, so not being nosy, but they've been caring, they care. So yeah, yeah, that's what I try and think anyway. Well, that's what someone told me to think.’

2. *‘Reconnected with meaningful activities of their past self’*

Due to deteriorating health, people with COPD over time typically adapt their activities that require exertion to reduce their symptom burden. For example, one participant discussed how they previously attended live football matches at the stadium with friends, whereas nowadays they are seated at home alone watching football on television (TV). Such lifestyle adaptations ultimately resulted in their social exclusion, including a loss of positive outcomes such as social connection (e.g., regular camaraderie of friends), identity development and maintenance (e.g., supporter of football), and physical activity. For several participants, this reconnection with their past self was largely a product of their re‐interpretation of the perceived physical barriers of COPD and associated sense of shame and fear of how others might perceive them. Engaging in the study provided participants with an essential ‘confidence boost’ in their capabilities to pursue opportunities that otherwise would have been missed (e.g., commencement of ocean swimming due to a neighbour's invitation ‐ for a participant who had been a competitive swimmer in high school).

Leanne was a 73‐year‐old woman who has stable COPD and met the criteria of a GOLD grade 1, and had a BODE score of 2. In the study Leanne found one of the barriers to reducing sedentary behaviour was a long‐held belief that being ‘old’ meant you were expected to be less active and inevitably sat more compared to when you were younger. Having a diagnosis of COPD or similar disease was part of getting older and slowing down. Leanne has strong memories of her grandmother and mother sitting during the day either reading, knitting or sleeping. This belief system was reinforced by her doctor who, as Leanne says, attributes all her health concerns to ‘getting older’ and, therefore, Leanne believed that there was nothing she could do to prevent or support herself with a diagnosis of COPD or other ailments. Leanne accepted the inevitability of being more sedentary as the only lifestyle option, as no one she knew challenged this belief. Leanne felt completely trapped as an ‘old self’ and was disconnected from the activities she used to enjoy in the past when she was younger because she did not know how to modify these activities as a person with COPD. Exposure to the study intervention gave Leanne new perspective on how getting older and having a diagnosis of COPD can mean that low level activity and sitting less are important key behaviours to improve your health and are obtainable at any stage of life. During the study, BCTs empowered Leanne to plan her daily study goals.

**Leanne:** ‘Just one thing that sounds silly, but I can now make my bed without having a rest. You know, I've always had to strip bed clothes and then do the washing. Normally, in the afternoon I've put the bottom sheet on, then a couple of hours later I put the top sheet on. I can now actually, when I do the washing, I can come back and make the bed all together. I can't remember the last time I made a bed all in one go. I'm going to be acting like a 50‐year‐old.’

3. *‘Reclaimed agency and autonomy’*

Due to disease‐specific barriers (e.g., dyspnoea, leg fatigue) people with COPD can experience social exclusion, feelings of low self‐worth and powerlessness, and inability to make positive decisions in their life. Participants in this study often felt no longer valued as a member of society and disconnected from the community due largely to decisions they had taken to deal with their disease specific barriers (e.g., taking early retirement, inability to participate in local activities) and lower economic status. Halted by low confidence and low self‐worth, participants felt they were unqualified to make important healthcare decisions about themselves. As one characteristic example, some participants waited for the clinician‐researcher to instruct them to activate their COPD action plan, despite experiencing worsening respiratory symptoms. Experiencing a release from a state of feeling trapped, together with reconnecting their former self, enabled participants to feel that they reclaimed some degree of agency and autonomy in their lives. Most participants found that the task of planning their own activities and times to break up their sedentary behaviour, transferred to making positive and meaningful decisions about their life more generally. As one example of this agency in action, one participant suggested that our face‐to‐face sessions occur at a local café by the beach rather than his house.

Another participant, Phil (81‐years‐old) who lives with his wife Diane, had stable COPD GOLD grade 3 and a BODE score of 3. Since Phil's diagnosis of COPD, he felt that important decisions such as managing his health had been removed from him, making him feel generally unsure and underconfident in making decisions.

**Phil:** I don't go to exercises (PRP) when it was too hot as it made me feel pretty dizzy and a bit shaky. Anyway, Diane kicked up such a fuss about me going along to exercises and driving, so I just gave in, it's easier just to go along with her as she doesn't let up until I actually lie down. My daughters are just as bad telling me I need to be careful in case I pass out and then I will be in hospital or worse.'

As part of the study Phil attended a focus group discussion with two other participants from the study, plus K.H. (experienced practitioner and supervisor) and the clinician‐researcher (facilitator). During the focus group discussion, the two participants talked about meeting the clinician‐researcher at a local café for their face‐to‐face sessions instead of meeting in their homes. At the end of the focus group discussion Phil asked me if he too could meet at a local café ‘like the others’ instead of his home. The previous face‐to‐face sessions were at Phil's home and this was familiar and a predictable setting where he spent most of his time as a retired man. The decision of Phil's to request a change of venue to meet for the face‐to‐face sessions was one of the first indicators that Phil was releasing himself from a state of being completely trapped, as in the past decisions were often made for him by his family.

**Phil:** ‘I would like to meet at a café shop overlooking the ocean near where I worked as a volunteer at the Marine Rescue group. I will meet you at the usual time at a table outside the café.’

See Appendix D for further examples of participants' experiences in the study.

## 5 Discussion

Engaging people with complex needs, such as those with COPD, in health behaviour change is a core component of chronic disease self‐management. In people with COPD, long held feelings of helplessness, powerlessness, and a sense of low self‐worth are reinforced by repeated difficult experiences often related to their COPD diagnosis (e.g., people with COPD being identified negatively with cigarette smoking). Qualitative research methods were used to explore the experiences of the participants throughout the study. Three important themes were generated from interviews and discussions with participants that revealed unanticipated benefits from the experimental intervention. The three themes were: (i) released from a state of feeling completely trapped, (ii) reconnected with meaningful activities of their past self and, (iii) reclaimed agency and autonomy.

Behaviour change techniques such as health education, action planning and goal setting allowed participants the opportunity to reinterpret their COPD diagnosis by being empowered to challenge long held beliefs of their inability to improve their life. Social support (emotional) from the clinician researcher provided participants with psychological support through listening, empathy and encouragement. The supportive environment created a platform to make meaningful change to achieve increased confidence in living with their disease and reclaiming agency and autonomy [23] so as to be able to inform family and friends of their limitations e.g., shortness of breath without fear of shame. Participants felt less trapped and were able to explore new experiences such as ocean swimming, and the ability to explain their diagnosis to family and others. Participants were beginning to have agency in decision making (e.g., choosing a café to meet with the clinician‐researcher) and feeling they were not trapped by their disease.

These themes of feeling completely trapped are similar to findings in a 2018 systematic review showing qualitative studies with facilitators and barriers to physical activity following PRP [24]. This study found, in participants with COPD, that the common barriers to participating in PRP were lack of self‐efficacy leading to feelings of self‐isolation or feelings of being trapped and powerlessness [24]. These are likely to be important target for healthcare professionals when facilitating people with COPD to engage in chronic disease self‐management.

Clinically, these findings suggest that addressing psychological capability – particularly low confidence in managing breathlessness – may be as important as targeting physical capacity when attempting to reduce sedentary behaviour in COPD. Participants described diminished confidence and reduced agency in relation to activity, within the context, the physiotherapist‐delivered BCTs (e.g., goal setting, action planning, graded tasks) appeared to strengthen perceived self‐efficacy and autonomy, despite no measurable change in ST. Together, these findings indicate that, for some individuals with COPD, barriers to reducing sedentary behaviour may reflect restraints in psychological capability, including low self‐efficacy and maladaptive illness beliefs, rather that solely physical limitation underscoring the need for interventions that explicitly target these mechanisms.

### 5.1 Limitations

These analyses were conducted by a team of three physiotherapists, all of whom have clinical experiences in the management of people with COPD. To address this potential problem with similar positionality, we worked with a fourth researcher, who was trained in psychology, but had no clinical experience with people with COPD. The person served as a ‘critical friend’ who questioned our interpretation of the data. A major limitation of the current study was the occurrence of the COVID‐19 pandemic. The effect of COVID‐19 pandemic on this single‐case experimental design study, was the suspension of recruitment of study participants as the hospital based PRPs were cancelled for 6 months. This limited the sample size available for this study. In Western Australia where the study was conducted the State borders were closed from April 2021 to March 2022 this allowed for far less ‘lock down’ restrictions periods compared to Europe and the United Kingdom. Western Australia had 2 weeks of ‘lock down’ restrictions within the year of the COVD‐19 pandemic where physical distancing and face masks were worn. However, this study's experimental intervention accommodated the restrictions of the COVID‐19 pandemic by the use of telehealth.

## 6 Conclusion

Although the study intervention did not change objective measures of ST, it provided a gateway for participants to achieve unanticipated benefits. Due to the clinician‐researcher spending significant time with participants during the study, participants' reflections and experiences were recorded revealing three themes representing the unanticipated benefits. These themes represented several changes in participant's lives that were of value to them. Importantly, harnessing these newfound benefits and beliefs in participant's lives may allow for further positive change in their health.

When evaluating any intervention, in addition to collecting objective measures, it is important for clinicians to consider unanticipated, but important changes such as the experience of people being able to re‐engage in valued life activities.

## Author Contributions

**Fiona Coll:** conceptualisation, methodology, data curation, visualisation, investigation, writing – original draft– reviewing and editing, project administration. **Vinicius Cavalheri:** conceptualisation, methodology, data curation, visualisation, investigation, writing – original draft, writing – reviewing and editing, supervision. **Daniel F. Gucciardi:** conceptualisation, methodology, data curation, visualisation, investigation, writing – reviewing and editing, supervision. **Kylie Hill:** conceptualisation, methodology, data curation, visualisation, investigation, writing – original draft, writing – reviewing and editing, supervision.

## Funding

The authors have nothing to report.

## Ethics Statement

The study was approved by the relevant Human Research Ethics Committees (HREC) to allow recruitment from four hospitals across Perth, Western Australia; Bentley Health Service approval number research governance system (RGS)3353, Joondalup Health Campus; approval number HREC1915, Rockingham General Hospital approval number RGS3353 and Royal Perth Hospital approval number RGS3353. Reciprocal ethics approval was obtained from the Human Research Ethics committee at Curtin University (HREC2019‐0429).

## Conflicts of Interest

The authors declare no conflicts of interest.

## Supporting information

Supporting File

## Data Availability

The data that supported the findings of the study are available from the corresponding author upon reasonable request.
