# Supplementary material for: A Qualitative Exploration of Unanticipated Effects Experienced By People With Chronic Obstructive Pulmonary Disease Who Participated in Interventions Designed to Reduce Sedentary Behaviour
Source: Health Expect. 2026 May 24;29(3):e70694. doi: 10.1111/hex.70694 (PMC13239887; doi:10.1111/hex.70694)
Supplement: Supplementary file 1 — Supporting File [file HEX-29-e70694-s001.docx]

**Appendix A**see Table A1

**Table A1 Standards of Reporting Qualitative Research (SRQR) checklist**

| **Title and abstract** |  | **Page numbers** |
| --- | --- | --- |
|  | **Title** - Concise description of the nature and topic of the study Identifying the study as qualitative or indicating the approach (e.g., ethnography, grounded theory) or data collection methods (e.g., interview, focus group) is recommended | 1 |
|  | **Abstract** - Summary of key elements of the study using the abstract format of the intended publication; typically includes background, purpose, methods, results, and conclusions | 1 |
| **Introduction** |  |  |
|  | **Problem formulation** - Description and significance of the problem/phenomenon studied; review of relevant theory and empirical work; problem statement | 3, 4 |
|  | **Purpose or research question -** Purpose of the study and specific objectives or questions | 4 |
| **Methods** |  |  |
|  | **Qualitative approach and research paradigm** - Qualitative approach (e.g., ethnography, grounded theory, case study, phenomenology, narrative research) and guiding theory if appropriate; identifying the research paradigm (e.g., postpositivist, constructivist/ interpretivist) is also recommended; rationale** | 9 |
|  | **Researcher characteristics and reflexivity -** Researchers’ characteristics that may influence the research, including personal attributes, qualifications/experience, relationship with participants, assumptions, and/or presuppositions; potential or actual interaction between researchers’ characteristics and the research questions, approach, methods, results, and/or transferability | 9 |
|  | **Context** - Setting/site and salient contextual factors; rationale** | 7, 8 |
|  | **Sampling strategy** - How and why research participants, documents, or events were selected; criteria for deciding when no further sampling was necessary (e.g., sampling saturation); rationale** | 4, 5 |
|  | **Ethical issues pertaining to human subjects -** Documentation of approval by an appropriate ethics review board and participant consent, or explanation for lack thereof; other confidentiality and data security issues | 4 |
|  | **Data collection methods** - Types of data collected; details of data collection procedures including (as appropriate) start and stop dates of data collection and analysis, iterative process, triangulation of sources/methods, and modification of procedures in response to evolving study findings; rationale** | 8, 9 |
|  | **Data collection instruments and technologies -** Description of instruments (e.g., interview guides, questionnaires) and devices (e.g., audio recorders) used for data collection; if/how the instrument(s) changed over the course of the study | 8, 9  Appendix B |
|  | **Units of study -** Number and relevant characteristics of participants, documents, or events included in the study; level of participation (could be reported in results) | 8, 9,  Appendix C |
|  | **Data processing** - Methods for processing data prior to and during analysis, including transcription, data entry, data management and security, verification of data integrity, data coding, and anonymization/de-identification of excerpts | 8, 9 |
|  | **Data analysis -** Process by which inferences, themes, etc., were identified and developed, including the researchers involved in data analysis; usually references a specific paradigm or approach; rationale** | 8, 9 |
|  | **Techniques to enhance trustworthiness -** Techniques to enhance trustworthiness and credibility of data analysis (e.g., member checking, audit trail, triangulation); rationale** | 8, 9 |
| **Results/findings** | **Synthesis and interpretation -** Main findings (e.g., interpretations, inferences, and themes); might include development of a theory or model, or integration with prior research or theory | 9 – 13, Appendix D |
|  | **Links to empirical data -** Evidence (e.g., quotes, field notes, text excerpts, photographs) to substantiate analytic findings | 9 – 13, Appendix D |
| **Discussion** |  |  |
|  | **Integration with prior work, implications, transferability, and contribution(s) to the field -** Short summary of main findings; explanation of how findings and conclusions connect to, support, elaborate on, or challenge conclusions of earlier scholarship; discussion of scope of application/generalizability; identification of unique contribution(s) to scholarship in a discipline or field | 13-15 |
|  | **Limitations -** Trustworthiness and limitations of findings | 15 |
| **Other** |  |  |
|  | **Conflicts of interest -** Potential sources of influence or perceived influence on study conduct and conclusions; how these were managed | 16 |
|  | **Funding -** Sources of funding and other support; role of funders in data collection, interpretation, and reporting | 1 |
|  | *The authors created the SRQR by searching the literature to identify guidelines, reporting standards, and critical appraisal criteria for qualitative research; reviewing the reference lists of retrieved sources; and contacting experts to gain feedback. The SRQR aims to improve the transparency of all aspects of qualitative research by providing clear standards for reporting qualitative research. **The rationale should briefly discuss the justification for choosing that theory, approach, method, or technique rather than other options available, the assumptions and limitations implicit in those choices, and how those choices influence study conclusions and transferability. As appropriate, the rationale for several items might be discussed together. |  |

**Appendix B: Example of interview guides used in the study**

**Participant semi-structured interview guide
The following questions will serve as a guide to interview participants prior to commencement of the behaviour change intervention of the study. The goal of the semi-structured interview is to gather information on potential barriers and enablers to reducing unbroken sedentary time for participants recruited in this study. The answers will be used to develop a personalised behaviour change intervention for the participant. These questions are based on areas of capability, opportunity, motivation and behaviour (COM-B) [17] linked to theoretical domains framework (TDF) domains [25].**

Prior to the commencement of the interview:

- Thank the participant for meeting the PhD candidate (co-ordinating investigator/ researcher)
- Explain the goal of the study and the purpose of the interview
- Provide and review essential information prior to commencement of the interview e.g. what is sedentary behaviour? (Standard information used prior to the initial Questionnaire the participant completed)
- Enquire if the participant has any questions, and to remind the participant that they are able to ask questions throughout the interview
- Inform the participant that the interview will be auto-recorded and how the audio recording will be treated (e.g., storage, analysis)

1. Tell me about yourself, describe what you do on a typical day during the week?

- Is what you do during the week different on the weekend?

1. What sort of work have you done over your life?

- What age did you retire?
- If you are still working when do you plan to retire?

1. Describe the neighbourhood in which you live?

- Do you feel safe in your neighbourhood?
- Are there people you could go walking with outside your home?
- Are there nearby parks or an area near where you live where you would like to walk? Are there seats in this park? Do you live near a bus stop? Do you live near a shopping centre? Are you able to drive to the shopping centre/ park/ area to walk?
- In your home environment is there space to do physical activity such as walking?
- Do you or would you walk in your home environment? Neighbourhood environment?
- Why?
- And why not?

1. Do you live on your own or with someone?

- Would you be able to nominate a significant person in your life that would accompany you to these behaviour change sessions? This person could be a family member, or close friend.
- Does this person help you with daily tasks? Probe with what, when, where, and how]
- How would you describe your significant person’s/ loved one’s attitude to activity?
- Their attitude to sedentary behaviour? e.g. sitting for long periods of time without getting up?
- What is your significant other’s/ loved one’s belief about activity and you?
- What about when you are unwell?

1. Are there other barriers to reducing sitting time?

- Situations that would stop you from standing up and/ or walking?
- Do other health conditions not your lung condition, make it difficult for you to reduce sitting time?
- Do other health conditions not your lung condition, make it difficult for you to increase light intensity physical activity?

1. From the previous Questionnaire you answered earlier, you nominated that when you were younger you participated in sports or physical activity (a lot/ some/ a little/ none/ don’t know) __________ as part of your life.

- Can you talk about these experiences?
- What stopped/ lessened continuing your participation with sports or physical activity?
- If sports or physical activity were not part of your life? What other things occupied your life?

1. Are you aware of different levels of exercise such as light intensity, moderate to vigorous intensity?

- Do you have goals around your daily physical activity?
- On average, how often do you engage in light intensity physical activity daily? Moderate intensity physical activity daily? Vigorous intensity physical activity daily?
- How do you feel during and after each type of activity?
- What helps or prevents you from doing more of each type of activity?
- Do you think there is a difference in the benefits of each category of physical activity?
- What do you think light intensity activity will achieve for you?
- Do you think there are reasons you should exercise at a light intensity level?
- What would motivate you to exercise? Is there anything that would help motivate you?
- How confident are you that you can do light intensity activity regularly, such as every 30 minutes?

1. How confident are you that you will be able to use the wearable technology like the Fitbit?

- How confident are you that you will be able to stand/ walk every 30 minutes during the day?
- In the last Questionnaire I gave you, you nominated _________________ as the greatest barrier (something that gets in the way) to sitting less during the day? How confident are you that ____________ will not stop you from standing/walking every 30 minutes?

Thank the participant for their time and sharing their experiences.

**Participant/ support person exit interview guide**

**The following questions will serve as a guide to interview participants and their support person about the experience of being part of the study and will assist with the process evaluation. The goal of the exit interview is for the participant and their support person to reflect on what was un/helpful during the study. These questions are based on aspects of capability, opportunity, motivation and behaviour (COM-B) linked to theoretical domains framework (TDF) domains [17, 25].**

Prior to the commencement of the interview:

- Thank the participant and support person for meeting the PhD candidate (co-ordinating investigator/ researcher) and being part of this study
- Explain the purpose of the interview
- Enquire if the participant/ support person has any questions, and to remind the participant that they are able to ask questions throughout the interview
- Inform the participant and support person that the interview will be auto-recorded

1. Can you tell me what you liked about being part of this study?

- What were the easiest factors to change in regards to reducing sedentary behaviour in your life?
- What were the hardest factors to change in regards to reducing sedentary behaviour in your life?

1. Can you tell me what you didn’t like about this study?
2. What did you think of the research team involved in the study?
3. Can you describe a typical day during the week?

- Describe a typical weekend day?

1. Have you learned any new (i) skills or (ii) information about physical activity and sedentary behaviour as part of this study?

- Why? or why not?
- What has changed in your life since the study?

1. Has your (i) home of (ii) neighbourhood environment changed since the start of the study?
2. Do you use your (i) home or (ii) neighbourhood environment in different ways because of taking part in this study? (What? Why? When? Where? How?)
3. Does your loved one help you to be more active/ break up sedentary time?

- (What? Why? When? Where? How?)

1. What is your loved one’s attitude to you engaging in physical activity and breaking up sedentary time?

- If it has changed, how so?

1. What motivates you to continue to reduce sedentary behaviour and replace it with light intensity physical activity?

- How important is reducing sedentary behaviour to you?
- Can you tell me why or why not?

1. How confident are you now in being able to break up sedentary time?
2. How confident are you in being able to maintain those changes in the future?
3. Can you nominate any existing barriers/ concerns you still have about reducing sedentary behaviour and increasing light intensity physical activity?

- Why do you think these situations have stopped you reducing sedentary behaviour and replacing it with light intensity physical activity?
- How confident are you that these barriers will reduce over time?
- Do other health conditions not your lung condition, make it difficult for you to reduce sedentary behaviour?
- Do other health conditions not your lung condition, make it difficult for you to increase light intensity physical activity?

1. Do you have any future goals in regards to being more active and less sedentary, since you have completed the study?

- How confident are you that you can achieve these goals?

1. Are there incentives for reducing sedentary behaviour?

- What motivates you to exercise?
- Is there anything that would help motivate you?
- How important is reducing sedentary behaviour to you?

Data are presented as mean (standard deviation) unless otherwise stated. BMI: body
mass index; FEV_1_: forced expiratory volume in 1 second; FVC: forced vital capacity. FEV1%pred: forced expiratory volume in 1 second percentage of the predicted value; GOLD: Global Initiative for Chronic Obstructive Lung Disease. CAT: COPD Assessment Test, mMRC: modified medical research Council dyspnoea scale; 6MWD: six-minute walk distance.

**Appendix C**

Table C1 Characteristics of participants

| Characteristic | n =10 |
| --- | --- |
| Age (year) | 70 (8) |
| Sex, n (%) male | 7 (70) |
| BMI (kg/m^2^) | 33 (9) |
| FEV_1_/FVC | 0.5 (0.2) |
| FEV_1_% pred | 53 (22) |
| GOLD Grade 1 FEV_1_% pred ≥ 80% GOLD Grade 2 FEV_1_% pred ≥ 50% to < 80% GOLD Grade 3 FEV_1_% pred ≥ 30% to < 50% GOLD Grade 4 FEV_1_% pred < 30% | 1  3  4  2 |
| CAT score (0 to 40) | 16 (7) |
| mMRC (0 to 4) | 2 (1) |
| 6MWD (m) | 390 (63) |
| Smoking pack years | 42 (15) |

| Characteristic | Male n=7 | Female n=3 |
| --- | --- | --- |
| Age (year) | 71 (7) | 66 (10) |
| BMI (kg/m2) | 29.60 (7.21) | 28.01 (8.32) |
| FEV_1_/FVC | 0.51 (0.22) | 0.53 (0.24) |
| FEV_1_% pred | 46.1 (20.2) | 61.7 (23.1) |
| CAT score (0 to 40) | 18 (8) | 13 (2) |
| mMRC (0 to 4) | 2 (1) | 2 (1) |
| 6MWD (m) | 371(69) | 399 (54) |
| Smoking pack years | 49 (11) | 24 (9) |

Data are presented as mean (standard deviation) unless otherwise stated. BMI: body
mass index; FEV_1_: forced expiratory volume in 1 second; FVC: forced vital capacity. FEV1%pred: forced expiratory volume in 1 second percentage of the predicted value; CAT: COPD Assessment Test; mMRC: modified medical research Council dyspnoea scale; 6MWD: six-minute walk distance.

Table C2 Characteristics of participants separated into male and female groups

**Appendix D: Further examples of the lived experiences of two participants in this study, where the three themes (reported in the manuscript) were supported by their quotes.**

***Case example 1 – Alex***

*‘Reconnected with meaningful activities of their past self’*

Clinician researcher asked Alex:

**Clinician researcher: ‘**Since being part of the study, do you use your home or neighbourhood environment differently?’.

Alex lived in a group of units where there were regular social events involving the residents and he found it difficult to remember to stand up after sitting for 30 minutes. As part of the study’s one-on-one face-to-face sessions, the clinician researcher recommended that Alex set a reminder vibrating alarm on his Smartphone for every 30 minutes to remind him to stand up from sitting between 5 and 8pm on a Thursday to Saturday evening. When the Smartphone alarm vibrated, Alex stood up to ‘stretch his legs or back’ or get himself or anyone else another drink to provide a legitimate reason to stand up. At the social events, the other residents were interested in Alex’s regular routine of moving from sitting to standing up every 30 minutes which gave him the opportunity to talk about the study he was involved in and therefore his diagnosis of COPD. In the past, Alex had felt shame and stigma with his diagnosis of COPD as he had been a long-term ex-smoker (60 pack-years) and, therefore, was reluctant to tell people due to the potential of a negative response. To his surprise, Alex found that the other occupants showed interest and support for him when prompted about his participation in this study.

Alex’s neighbour Patrick was supportive of Alex being part of the study which helped him feel worthy and valued by others in his community, something which he had not felt for some time.

**Alex:** ‘And he (Patrick a neighbour) sort of cares what yeah, it takes us (Alex) seriously, with what I’ve got (COPD) we're worried about what’s up, he understands that what we (the study) were doing was helping. Right. So, he (Patrick) used to say, “Get up, stand up, stand up.”’

Alex and his neighbour Patrick had attended the same high school, and both had also trained as butchers; due to this commonality, they formed a close friendship. Patrick instigated the idea of creating a gym to support Alex in being more active and sitting less. Alex and Patrick constructed the gym at the back of the units where there were two vacant car bays side by side. Alex has used the gym intermittently.

The friendship and support of Alex’s high school friend who also lived in the same block of units has been a major catalyst of motivation for Alex throughout the study. This friendship between Alex and Patrick helped Alex reconnect with elements of his former self via social connectiveness and nostalgia which resulted in constructing a gym together.

**Alex**: ‘We're starting a gym out the back. Okay, that's the one that was the biggest change because of the study, because the next door neighbour (Patrick), he got on board and he says, “we share a carport out the back that we don’t use. So, all set up with an exercise bike and weights.”’.

*‘Released from a state of feeling completely trapped’*

Robert was another resident who lived at the units and was a regular ocean swimmer. Robert encouraged Alex to walk to the beach and to try ocean swimming with him, because at school Alex had been a competitive swimmer and water polo player. Alex found the commitment of meeting Robert each morning to swim at the beach was a strong motivator and source of support. Due to Alex increasing his swimming distance therefore he needed more time, he went down the beach earlier, and therefore met Robert for a coffee at the end of their swimming. Over the time of the study, Alex reported that he had reduced the time it took him to walk to the beach and increased the distance he swam.

During the study, Alex was able to accept the challenge of walking and swimming at the beach, something which would have been impossible previously when feeling trapped by his fear of shortness of breath. This experience was also an example of ‘reclaiming agency and autonomy’ as Alex was committed to meet Robert despite fearing that he may become short of breath or unable to complete the task. Alex registered Robert’s support in helping him succeed at this task without feeling that he was unworthy or a burden.

**Alex:** ‘And as I'm walking down (the beach) with Robert, the 76-year-old, after the first three weeks, he walked slow, because he (normally) walks fast, but he would walk slow for me. And then on the third week, we're walking down the block and he says to me, “Alex (he says), I'm (Robert) not slowing down anymore”. I was now walking at his pace.’

***Case example 2 – Leanne***

*‘Released from a state of feeling completely trapped’*

Leanne felt at times she was underappreciated by the players at her local Lawn Bowls club as they did not acknowledge her hard work of organising lawn bowls events despite Leanne feeling tired with her diagnosis of COPD. Leanne found her shortness of breath to be stressful, especially when playing lawn bowls as she felt she had to struggle alone and not let people know of her limitations. Leanne believes if she told people about her shortness of breath they may dismiss her health concerns as ‘something you have to live with’, therefore making her feel inconsequential. Leanne’s sense of ‘feeling alone’ with her COPD diagnosis became less influential in her experience, largely because of her attendance at PRP with other people who had a diagnosis of COPD, and her regular contact with the clinician researcher Importantly, Leanne was receptive to health education as part of BCT and was able to improve her understanding of her COPD diagnosis. The implementation of behaviour change supported a reinterpretation of Leanne’s COPD diagnosis releasing her feelings of being completely trapped, as she could pace her activity and, therefore, her shortness of breath was more manageable and less stressful.

**Clinician researcher:** ‘Can you tell me what you liked about being part of the study?’

**Leanne:** ‘Yes, I found that really informative going to those classes. Because I realised that there’s all different levels of health, chest and lung problems. Some people are a lot better off than others. And I think it made me realise that I need to keep exercising so that I don’t end up worse off. Okay, you sort of made me very aware of that. I think it's all made me aware of what I should be doing to improve my health. I think when you're first diagnosed with COPD, you just think, oh, no, I've got this and I'm stuck with it for good, you don't think that you can ease it at all. But, when this study came along, and you find out or by doing exercises, and by doing that, you're not going to cure it (COPD), but you can improve your life. So, I think that's been a huge thing for me, and I thank you for that.’

*‘Reclaimed agency and autonomy’*

The empowerment of learning about her disease and how she can help herself assisted Leanne in reclaiming agency and autonomy and giving her confidence to inform others of her limitations of shortness of breath.

**Leanne:** ‘But I wasn’t alone. I didn’t think anyone else had COPD until I started the study. I was just alone a person with COPD and that I struggled, struggled every day. And then I found that my struggling, it was quite awful because of the breathing or lack of breathing. I think that was an important part of the study to find that out. Because I don’t think beforehand that I ever said anything about COPD. When I was struggling, people thought: “Oh she’s struggling or whatever.” “But, now if I am struggling with my breathing I just say”:

“it’s really humid today, I’m struggling to breathe. So, I will be sitting down. I will not be walking a lot”. I will do what I have to do and I’m happy to tell people and I wasn’t able to do beforehand (before the study).’
